# Pleural Metastatic Melanoma With Recurrent Malignant Pleural Effusions

**DOI:** 10.7759/cureus.64366

**Published:** 2024-07-11

**Authors:** Sonal Prasad, Jay Xiong, Jasleen Kaur

**Affiliations:** 1 Internal Medicine, St. Joseph's Medical Center, Stockton, USA; 2 Internal Medicine, St. Joseph’s Medical Center, Stockton, USA

**Keywords:** decortication, pleural melanoma, metastatic melanoma, malignant pleural effusions, pleural metastatic melanoma

## Abstract

Pleural metastatic melanoma is rare, and associated malignant pleural effusions are even rarer. We present a case of pleural metastatic melanoma with recurrent malignant pleural effusions. The initial diagnosis showed no metastatic disease, and the patient underwent resection and received a year of immunotherapy for localized disease. However, two years later, the patient presented with pleural metastatic melanoma with unresolving malignant pleural effusions requiring an indwelling pleural catheter and eventually, thoracotomy with decortication. Clinicians should have a high index of suspicion for pleural metastatic melanoma in the setting of recurrent pleural effusions, even though it is a rare occurrence.

## Introduction

Melanoma is the third most common skin cancer and the fifth most common cancer in males and females. The 2018 World Health Organization (WHO) Classification of Melanoma separates melanomas into three distinct categories: melanoma that is associated with solar damage, melanoma that is not associated with solar damage, and nodular melanomas. Environmental, genetic, and immunological factors play an important role in melanoma. If it is diagnosed early, survival rates are as high as 94%. If metastasis is present, the prognosis is poor. Melanoma can spread to the liver, bones, brain, or lungs. The lung is a common site of metastasis, and when lung metastasis is present, the most common cause of death is respiratory failure. The incidence rate of metastatic melanoma is about 0.9 per 100,000 [1]. Pleural metastatic melanoma is rarer and not commonly reported. Additionally, only 2% of the patients with thoracic metastatic melanoma present with malignant pleural effusions [2]. Differentiating between primary pleural neoplasm and pleural metastatic melanoma can be tricky given similar presentations. Prompt diagnosis by pleural biopsy is essential for initiating treatment early. We report a case of a patient with a history of right first toe melanoma status post resection and immunotherapy who presented with shortness of breath. She was later found to have a pleural metastatic melanoma with massive recurrent pleural effusions.

## Case presentation

A 77-year-old female with a medical history of hypertension, chronic kidney disease stage III, osteoarthritis, degenerative disc disease, and right first toe melanoma status post resection and immunotherapy presented with shortness of breath. She reported gradually worsening shortness of breath for the last two weeks. Over the past few days, she was short of breath while at rest, which prompted her to come to the emergency department. She denied chest pain, palpitations, sore throat, body aches, fever, chills, nausea, vomiting, or pedal edema. She denied using oxygen at baseline.

**Figure 1:**
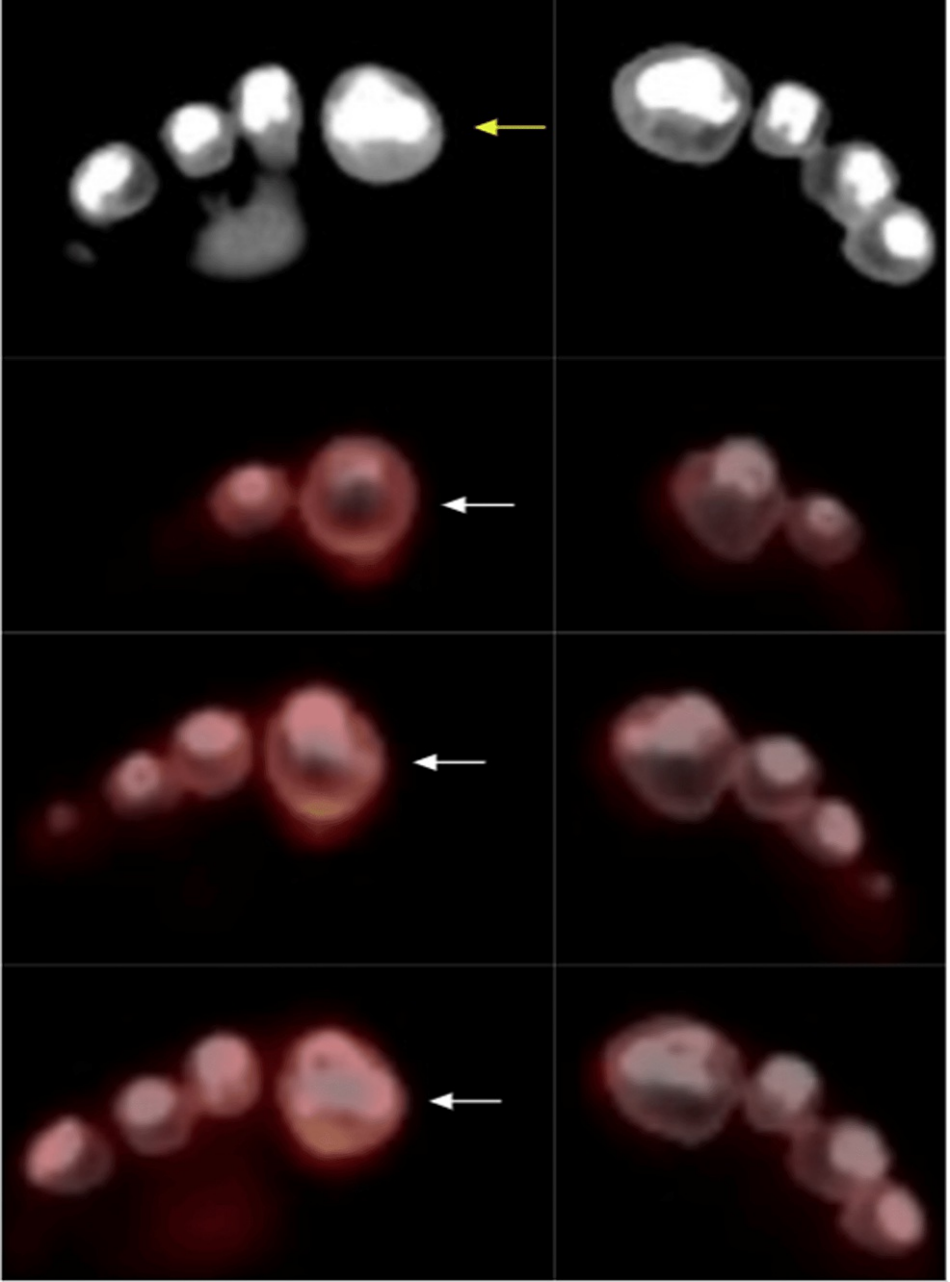
Right first toe focal moderate level increased FDG localization (white arrows) without abnormal anatomical correlate (yellow arrow). FDG: Fluorodeoxyglucose

Relevant past medical history included that about two years ago, the patient had a biopsy of her right great toe that revealed a melanotic lesion showing atypical dermal melanocytic proliferation suspicious for melanoma. Tumor cells were strongly positive for S100, SOX10, MITF, and HMB45. Positron emission tomography-computed tomography scan revealed right first toe focal moderate level increased fluorodeoxyglucose (FDG) localization correlating with melanoma only in the right great toe (Figure 1) with no involvement of regional sites, local lymph nodes, or distant metastatic disease. She underwent a right great toe amputation. Final pathology showed invasive melanoma with a distal thickness of 3 mm without ulceration. Metastatic melanoma was noted in one of two sentinel lymph nodes removed with metastatic melanoma. The final pathological classification was T3a, pN1a, M0. The patient was started on adjuvant immunotherapy with nivolumab 480 mg every four weeks. She received at least nine months of treatment before developing inflammatory side effects from the medication. Her regimen was adjusted to nivolumab 240 mg every two weeks. She received one year of immunotherapy.

**Figure 2:**
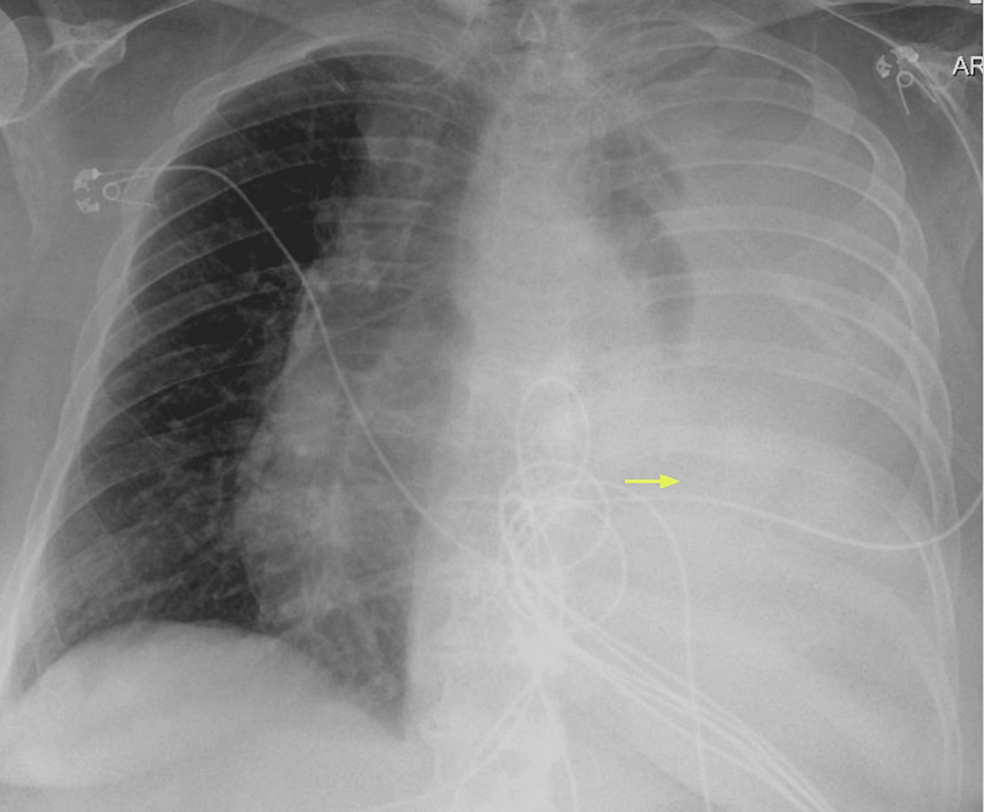
There is near complete opacification of the left hemithorax with large left pleural effusion (yellow arrow).

**Figure 3:**
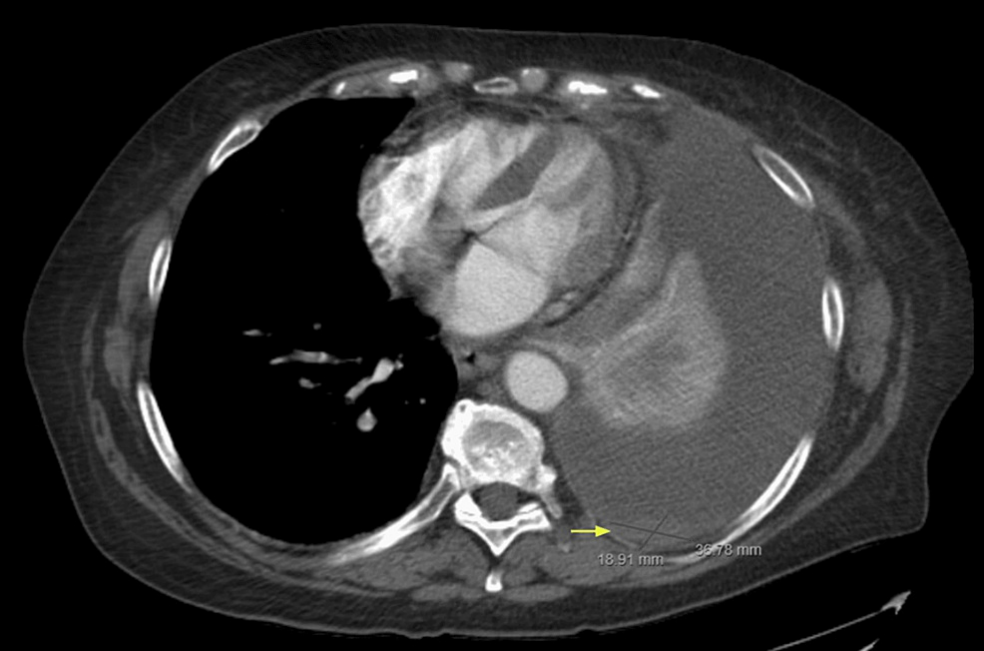
Pleural-based mass is seen involving the left lung measuring up to 3.7 x 1.9 cm (yellow arrow) with large left pleural effusion occupying nearly the entirety of the left lung.

On presentation, vital signs were significant for a respiratory rate of 26 breaths/minute and the patient required three liters of oxygen via nasal cannula. Physical examination was notable for diminished breath sounds over the left lung. Chest radiography revealed near complete opacification of the left hemithorax and large left pleural effusion (Figure 2). The computed tomography angiography (CTA) of the chest showed multiple pleural-based masses involving the left lung measuring up to 3.7 x 1.9 cm with large left pleural effusion occupying nearly the entirety of the left lung and multiple nodules throughout the right lung measuring up to 1.2 cm (Figure 3). The patient received two thoracenteses with a total of three liters of dark red colored fluid removed. Per light’s criteria, pleural fluid analysis was positive for exudative effusion. Despite the thoracenteses, the pleural effusion continued to reaccumulate. Therefore, the patient received an indwelling pleural catheter for continuous drainage. She also had a port placed and was discharged home with plans to start on a combination dual therapy of nivolumab and relatlimab for metastatic melanoma.

**Figure 4:**
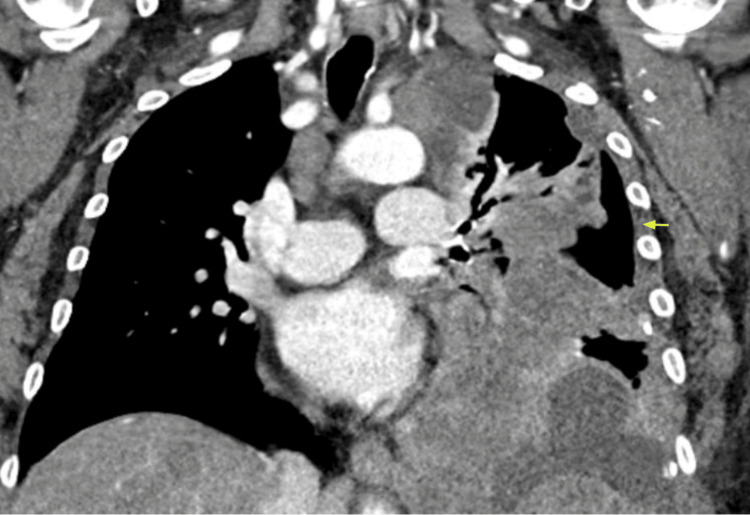
Complex loculated left pleural effusion (yellow arrow).

**Figure 5:**
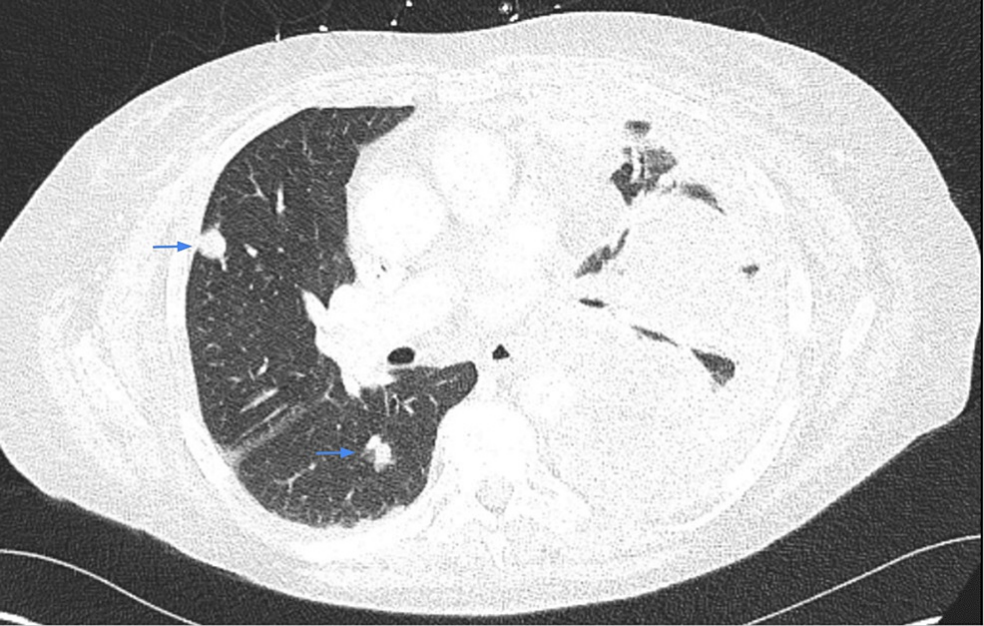
Multiple pulmonary nodules (blue arrows).

One month later, the patient re-presented to our hospital with shortness of breath. On presentation, she required five liters of oxygen via nasal cannula. CTA chest showed multiple enlarged mediastinal and hilar lymph nodes, interval worsened metastatic large complex loculated left pleural effusion, large pleural metastases, and numerous pulmonary nodules (Figure 4 and Figure 5). Per history, the indwelling pleural catheter was not draining for the past two weeks prior to presentation. There was a concern that the indwelling pleural catheter was not draining but the patient was not a candidate for tissue plasminogen activator/dornase alfa treatment given the patient's history of hemorrhagic pleural effusion. Interventional radiology was consulted but were unable to aspirate any fluid due to the severe loculations. Cardiothoracic surgery was consulted for alternative treatment options. The patient underwent left thoracotomy with decortication and left chest tube placement. Left pleural tissue was sent for pathology which showed a population of malignant cells that were pleomorphic and anaplastic. Controlled immunohistochemistry demonstrated these cells to be S100 positive and HMB45 positive, supporting the clinical impression of metastatic melanoma. Eventually, the chest tube was removed, and the patient was weaned off to room air. She was discharged with a plan for salvage therapy for advanced melanoma with treatment consisting of dabrafenib and trametinib.

## Discussion

Melanoma is largely responsible for deaths related to skin cancer. Lesions suspicious for melanoma can be identified with common features that are listed in the ABCDE mnemonic: asymmetry of shape, border irregularity, color variation, diameter greater than 6 mm, and evolution of the lesion [3]. Melanoma can spread locally, regionally, and distantly [1]. To make the diagnosis of melanoma, a full-thickness excision biopsy is generally necessary. Melanoma staging is done based on the Tumor, Nodes, and Metastases classification. This considers the tumor thickness and if there is ulceration present or not, whether there is involvement of regional lymph nodes and non-nodal regional sites, and if there are distant metastases [4]. The National Comprehensive Cancer Network (NCCN) has guidelines for the treatment of malignant melanoma based on this classification.

Metastatic melanoma of the lung is common, but it only comprises about 5% of all secondary pulmonary malignancies. Additionally, metastasis to the pleura is rare and it is uncommon to have malignant pleural effusions. Only 2% of patients with thoracic metastases have pleural effusions [2,5]. The study by Chen et al. reported three patients (2%) with malignant pleural effusions in the setting of metastatic melanoma to the thorax [6]. Pleural metastatic melanoma can present as pleural thickening or pleural effusion. When present, the pleural effusion can be unilateral or bilateral, small or large, and can be black in color if there are melanocytes present. Malignant pleural effusions are expected to be exudative and about 60% of the time can be diagnosed on pleural fluid cytology [7].

Recurrent malignant pleural effusions usually signify advanced disease. Therefore, the management of malignant pleural effusions depends on the specific clinical scenario with factors such as patient age, life expectancy, response to cancer therapy, and symptomatic relief. Therapeutic options include repeated thoracentesis, indwelling pleural catheter, pleurodesis, decortication, and chemotherapy [8].

Our patient presented with recurrent large pleural effusions, dark red in color indicating hemorrhagic effusion. The pleural fluid was exudative making malignant pleural effusion top on our differential list and subsequently, a left pleural peel biopsy confirmed the diagnosis of metastatic melanoma. Despite the placement of the left-sided indwelling pleural catheter, our patient presented again with left-sided loculated pleural effusion. Ultimately, the patient required thoracotomy with decortication.

When our patient was initially diagnosed with cutaneous malignant melanoma, the stage was T3a, pN1a, M0 which was treated with resection and Nivolumab per the NCCN guidelines. Despite adequate treatment, the disease progressed, and she was found to have distant metastases. Malignant melanoma is an aggressive skin cancer and pleural metastasis is considered a poor prognostic factor. In our patient, pleural involvement occurred two years after the diagnosis of malignant melanoma of the right great toe. Per the NCCN guidelines, the patient was started on systemic therapy with dabrafenib and trametinib. Our case is unique in that pleural metastatic melanoma is very rare and there are only a few reports of it presenting as massive recurrent pleural effusions.

## Conclusions

In conclusion, distant metastatic melanoma carries an overall poor prognosis. The rarity of metastatic pleural melanoma presenting as malignant pleural effusion may disguise metastatic disease as a primary lung or pleural tumor. Early identification is important because the most common cause of death in metastatic melanoma is respiratory failure secondary to lung or pleural involvement. A high index of suspicion for uncommon findings such as recurrent malignant pleural effusions in a patient with a history of melanoma can lead to prompt treatment. This can lead to improved overall outcomes. Depending on patient preference and survival expectancy, management can be aimed toward symptomatic relief as palliative treatment versus aggressive treatment for cure. 
